# Supplementary material for: HIVprotI: an integrated web based platform for prediction and design of HIV proteins inhibitors
Source: J Cheminform. 2018 Mar 9;10:12. doi: 10.1186/s13321-018-0266-y (PMC5845081; doi:10.1186/s13321-018-0266-y)
Supplement: Supplementary file 2 — Additional file 2. Source code of HIVProtI web server. [file 13321_2018_266_MOESM2_ESM.zip › HIVprotI_Source-code/hivprotI/weka-3-6-6/doc/weka/gui/arffviewer/package-tree.html]

weka.gui.arffviewer Class Hierarchy


---


|  |  |  |
| --- | --- | --- |
| **Overview** \| **Package** \| Class \| **Tree** \| **Deprecated** \| **Index** \| **Help** \| **Weka's home** | |  |
| **PREV**   **NEXT** | **FRAMES**    **NO FRAMES**     **All Classes** |  |


---


## Hierarchy For Package weka.gui.arffviewer

**Package Hierarchies:**: All Packages

---

## Class Hierarchy

- java.lang.Object
  - javax.swing.table.AbstractTableModel (implements java.io.Serializable, javax.swing.table.TableModel)
    - weka.gui.**SortedTableModel** (implements javax.swing.event.TableModelListener)
      - weka.gui.arffviewer.**ArffSortedTableModel** (implements weka.core.Undoable)
    - weka.gui.arffviewer.**ArffTableModel** (implements javax.swing.table.TableModel, weka.core.Undoable)- java.awt.Component (implements java.awt.image.ImageObserver, java.awt.MenuContainer, java.io.Serializable)
        - java.awt.Container
          - javax.swing.JComponent (implements java.io.Serializable)
            - javax.swing.JLabel (implements javax.accessibility.Accessible, javax.swing.SwingConstants)
              - javax.swing.table.DefaultTableCellRenderer (implements java.io.Serializable, javax.swing.table.TableCellRenderer)
                - weka.gui.arffviewer.**ArffTableCellRenderer**
              - javax.swing.JPanel (implements javax.accessibility.Accessible)
                - weka.gui.arffviewer.**ArffPanel** (implements java.awt.event.ActionListener, javax.swing.event.ChangeListener, java.awt.event.MouseListener, weka.core.Undoable)- weka.gui.arffviewer.**ArffViewerMainPanel** (implements java.awt.event.ActionListener, javax.swing.event.ChangeListener)
                - javax.swing.JTable (implements javax.accessibility.Accessible, javax.swing.event.CellEditorListener, javax.swing.event.ListSelectionListener, javax.swing.event.RowSorterListener, javax.swing.Scrollable, javax.swing.event.TableColumnModelListener, javax.swing.event.TableModelListener)
                  - weka.gui.arffviewer.**ArffTable**
            - java.awt.Window (implements javax.accessibility.Accessible)
              - java.awt.Frame (implements java.awt.MenuContainer)
                - javax.swing.JFrame (implements javax.accessibility.Accessible, javax.swing.RootPaneContainer, javax.swing.WindowConstants)
                  - weka.gui.arffviewer.**ArffViewer** (implements java.awt.event.WindowListener)
        - weka.gui.arffviewer.**Messages**

---


|  |  |  |
| --- | --- | --- |
| **Overview** \| **Package** \| Class \| **Tree** \| **Deprecated** \| **Index** \| **Help** \| **Weka's home** | |  |
| **PREV**   **NEXT** | **FRAMES**    **NO FRAMES**     **All Classes** |  |


---
